# Management of adverse events associated with bosutinib treatment of chronic-phase chronic myeloid leukemia: expert panel review

**DOI:** 10.1186/s13045-018-0685-2

**Published:** 2018-12-27

**Authors:** Jorge E. Cortes, Jane F. Apperley, Daniel J. DeAngelo, Michael W. Deininger, Vamsi K. Kota, Philippe Rousselot, Carlo Gambacorti-Passerini

**Affiliations:** 1 University of Texas MD Anderson Cancer Center, 1515 Holcombe Blvd, Houston, TX 77030 USA; 2 Hammersmith Hospital, London, UK; 3 Department of Medical Oncology, Dana-Farber Cancer Institute, Harvard Medical School, Boston, MA USA; 4 University of Utah Huntsman Cancer Institute, Salt Lake City, UT USA; 5 Winship Cancer Institute of Emory University, Atlanta, GA USA; 6 Versailles University, Versailles, France; 7 University of Milano-Bicocca, Milan, Italy

**Keywords:** Tyrosine kinase inhibitor, Bosutinib, Chronic myeloid leukemia, Dosing strategies, Adverse events

## Abstract

Bosutinib, a BCR-ABL1 tyrosine kinase inhibitor (TKI), has been available for several years as a treatment for chronic-, accelerated-, and blast-phase chronic myeloid leukemia (CML), for patients with resistance or intolerance to prior therapy. In 2017, the BFORE trial demonstrated efficacy of bosutinib as first-line treatment in adult patients with newly diagnosed chronic-phase chronic myeloid leukemia (CP-CML). The most common adverse events (AEs) of any grade in bosutinib-treated patients in BFORE were diarrhea, nausea, thrombocytopenia, increased alanine aminotransferase, and increased aspartate aminotransferase, consistent with the most commonly reported AEs in earlier studies. To balance the efficacy and tolerability of treatment to optimize patient adherence with medications, treating physicians commonly use various strategies such as initiating treatment at a lower dose, dose reduction, or dose interruption, depending on the type and severity of the AEs and the clinical setting. In light of the recent data from first-line treatment, an expert panel of hematologists reviewed management strategies for the use of bosutinib in treatment of CP-CML and made the recommendations reported here. Although the panel focused on first-line treatment, the principles can be for the most part extended to bosutinib use in later lines of treatment. Recommendations include advice regarding prophylaxis and management for diarrhea. The panel also considered optimum timing for referral to a specialist for specific AEs. Across the commonly occurring AEs, the panel highlighted the importance of education and communication with patients about anticipated AEs.

## Background

Bosutinib is an oral, second-generation BCR-ABL1 tyrosine kinase inhibitor (TKI). Bosutinib was first approved in 2012 for treatment of chronic-, accelerated-, and blast-phase CML in patients previously treated with one or more TKIs and for whom imatinib, nilotinib, and dasatinib are not considered appropriate treatment options or in patients resistant or intolerant to prior therapy, according to guidelines of the European Medicines Agency (EMA) and US Food and Drug Administration (FDA), respectively [1, 2]. Bosutinib was then approved (in 2017 in the USA and 2018 in the European Union) for first-line (1L) treatment of adult patients with newly diagnosed chronic-phase (CP) chronic myeloid leukemia (CML) [1, 2].

Bosutinib treatment for patients with CML has been studied in three large trials. For second-line and subsequent-line (≥ 2L) treatment, a phase 1/2 study studied bosutinib 500 mg once daily (QD) in patients with imatinib-resistant or imatinib-intolerant CML [3, 4]. The study analyzed two groups based on their treatment history: a 2L cohort of patients previously treated with imatinib only, and ≥ 3L cohort of patients previously treated with imatinib plus dasatinib and/or nilotinib [3, 4]. For 1L treatment, bosutinib 500 mg QD was compared with imatinib 400 mg QD in the BELA trial [Bosutinib Efficacy and safety in newly diagnosed chronic myeloid LeukemiA] [5]. Subsequently, bosutinib 400 mg QD was also studied as a 1L treatment, in the BFORE study [Bosutinib trial in First-line chrOnic myelogenous leukemia tREatment] [6].

In the ≥ 2L phase 1/2 study, 31% of patients in the 2L cohort achieved major cytogenetic response at 24 weeks with bosutinib 500 mg QD, and 86% of patients achieved complete hematologic remission at the longer-term follow-up (after a median of 24.2 months) [3]. At 2 years, progression-free survival was 79% and overall survival 92% [3]. Efficacy was durable, with long-term follow-up after 5 years showing 60% had achieved major cytogenetic response and overall survival was 84% [7]. In the ≥ 3L cohort, 32% of patients achieved major cytogenetic response, 24% complete cytogenetic response, and 73% complete hematologic response [4]. At 2 years, progression-free survival was 73% and overall survival was 83% [4]. Long-term follow-up at 4 years found a major cytogenetic response rate of 40% and overall survival of 78% [8].

In 1L treatment, improvements in various secondary endpoints in BELA suggested potential for bosutinib, but found similar rates of complete cytogenetic response at 12 months (the primary endpoint of the study; 70% with bosutinib 500 mg QD and 68% with imatinib 400 mg QD, *P* = 0.601) [5], and thus bosutinib did not obtain regulatory approval for 1L use at that time. However, analysis of BELA data showed a higher discontinuation rate at 12 months with bosutinib, most commonly for adverse events (AEs) and frequently without management of such AEs prior to discontinuation, suggesting a lower bosutinib dose and proper management of AEs might have allowed patients to stay on treatment for longer and in turn, experience clinical benefit [5]. A reduced starting dose (400 mg QD bosutinib) did indeed demonstrate the efficacy of 1L bosutinib versus 400 mg QD imatinib in the BFORE study, which compared initial therapies in patients with CP-CML, with primary efficacy analyses conducted in the modified intent-to-treat population (Philadelphia chromosome–positive patients with typical *BCR-ABL1* transcript types) [6]. The primary endpoint, major molecular response rate at 12 months, was 47.2% versus 36.9% for bosutinib versus imatinib (*P* = 0.02), and the rate of complete cytogenetic response by 12 months was 77.2% versus 66.4% (*P* = 0.0075) [6]. In the safety population, the most common treatment-emergent AEs of any grade (i.e., incidence ≥ 20%) in the bosutinib group were diarrhea (70.1%), nausea (35.1%), thrombocytopenia (35.1%), increased alanine aminotransferase (ALT; 30.6%), and increased aspartate aminotransferase (AST; 22.8%) [6]. AEs were consistent with the known safety profile of bosutinib as demonstrated in earlier studies, including 2L and subsequent-line treatment, as well as the BELA trial [3–5, 8–10].

Dose adjustments are commonly required with all TKIs in patients with CML and can be used over the course of treatment to achieve a proper balance of efficacy and safety [11]. The benefits and risks of different TKIs for the treatment of patients with CML have been discussed in previous publications [12, 13]. In this manuscript, we summarize the recommended management strategies employed in the use of bosutinib in 1L treatment and after prior TKI therapy (≥ 2L) for CP-CML.

## Setting and methods

An expert panel meeting was held on October 14, 2017 in Estoril, Portugal. Seven hematologists from different academic centers in the USA and Europe participated; all with extensive experience of treating CML, including use of bosutinib. To reach a consensus during the meeting, the expert group reviewed and discussed the responses to a number of pre-meeting questions on dosing strategies and management of AEs with bosutinib. The group discussed treatment management and guidelines, the dosing strategies they apply in practice, the patient characteristics that influence their decisions, and how they manage AEs when using bosutinib in CP-CML. The panel’s discussions during the meeting were drafted as a manuscript, and following critical review by all experts, their final recommendations are presented below.

## AEs experienced by patients with CML during treatment with TKIs

TKIs are the standard treatment for CML, and of the five widely available TKIs, four—imatinib [14], nilotinib [15], dasatinib [16], and bosutinib [1]—are approved for 1L treatment of CML (Table 1). Ponatinib is more typically used in the 3L setting; in the USA, it is approved for use in patients with CML who have the T315I mutation of *BCR-ABL1* or for whom no other TKI therapy is appropriate [17] and the EMA additionally restricts treatment to patients with the T315I mutation, or patients who are resistant to dasatinib or nilotinib, or intolerant to dasatinib or nilotinib and where subsequent imatinib treatment is not clinically appropriate [18]. Although the various TKIs have AEs common to the class, frequencies vary, and different TKIs also have AEs unique or more common with a specific agent, and frequently have specific warnings about particular AEs (Table 1).

**Table 1:** TKIs used for the treatment of patients with CP-CML: overview of US prescribing information*

TKI	Mechanism of action	Indications in adults with CP-CML (as of September 2018)	Most frequently reported AEs	Warnings and precautions
Bosutinib [1]	• Inhibits BCR-ABL1 and SRC family (including SRC, LYN, and HCK) kinases	• 1L • ≥ 2L in patients with resistance or intolerance to prior therapy	• Incidence ≥ 20%: diarrhea, nausea, thrombocytopenia, rash, vomiting, abdominal pain, anemia, pyrexia, liver test abnormalities, fatigue, cough, headache, and edema	• No black-box warnings • Fetal harm • GI toxicity: diarrhea, nausea, vomiting, abdominal pain • Myelosuppression: thrombocytopenia, anemia, neutropenia • Hepatic toxicity: one case of drug-induced liver injury (defined as concurrent elevations in ALT or AST ≥ 3 × ULN with total bilirubin > 2 × ULN and alkaline phosphatase < 2 × ULN) • Fluid retention: may manifest as pericardial effusion, pleural effusion, pulmonary edema, and/or peripheral edema • Renal toxicity: decline in estimated glomerular filtration rate
Dasatinib [16]	• Inhibits BCR-ABL1, SRC family (SRC, LCK, YES, and FYN), c-KIT, ephrin (EPH) receptor A2, PDGFRβ kinases	• 1L • ≥ 2L in patients with resistance or intolerance to prior therapy	• Incidence ≥ 15%: myelosuppression, fluid retention events (with pleural effusion occurring in 28% during long-term follow-up), diarrhea, headache, skin rash, hemorrhage, dyspnea, fatigue, nausea, and musculoskeletal pain	• No black-box warnings • Fetal harm • Myelosuppression: thrombocytopenia, neutropenia, anemia • Bleeding-related events (mostly associated with severe thrombocytopenia): central nervous system, GI hemorrhages • Fluid retention: sometimes severe, including pleural effusions • QT prolongation • Cardiac dysfunction, including ischemic events, cardiac-related fluid retention, arrhythmia, and palpitations • Pulmonary arterial hypertension • Severe dermatologic reactions • Tumor lysis syndrome
Imatinib [14]	• Inhibits BCR-ABL1, stem cell factor, c-KIT, PDGFR kinases	• 1L (follow-up limited to 5 years) • ≥ 2L after failure of interferon-alpha therapy	• Incidence ≥ 30%: edema, nausea, vomiting, muscle cramps, musculoskeletal pain, diarrhea, rash, fatigue, abdominal pain	• No black-box warnings • Fetal harm • Edema and severe fluid retention • Anemia, neutropenia, thrombocytopenia • Severe congestive heart failure, LV dysfunction • Severe hepatotoxicity • Grade 3/4 hemorrhage and GI perforations • Cardiogenic shock/LV dysfunction in patients with conditions associated with high eosinophil levels • Bullous dermatologic reactions • Hypothyroidism • Tumor lysis syndrome • Renal toxicity • Motor vehicle accidents
Nilotinib [15]	• Inhibits BCR-ABL1, PDGFR, c-KIT, colony stimulating factor-1 receptor, discoidin domain receptor-1 kinases	• 1L • ≥ 2L in patients with resistance or intolerance to prior therapy that included imatinib	• Incidence ≥ 20% (non-hematologic): nausea, rash, headache, fatigue, pruritus, vomiting, diarrhea, cough, constipation, arthralgia, nasopharyngitis, pyrexia, night sweats • Most common hematologic: thrombocytopenia, neutropenia, anemia	• Black-box warning for QT prolongation and sudden death. Do not administer in patients with hypokalemia, hypomagnesemia, or long QT syndrome; avoid concomitant drugs known to prolong QT interval and strong CYP3A4 inhibitors; avoid food 2 h before and 1 h after dose • Fetal harm • Myelosuppression: neutropenia, thrombocytopenia, anemia • Cardiac and arterial vascular occlusive events • Pancreatitis, elevated serum lipase • Hepatotoxicity: elevations in bilirubin, AST/ALT, alkaline phosphatase • Electrolyte abnormalities: hypophosphatemia, hypokalemia, hyperkalemia, hypocalcemia, hyponatremia • Tumor lysis syndrome • Hemorrhage • Total gastrectomy (removal of the entire stomach) • Fluid retention: pericardial effusion, pleural effusion, severe fluid retention • Treatment discontinuation; monitor frequently for typical BCR-ABL transcripts
Ponatinib [17]	• Inhibits ABL and T315I mutant ABL, SRC family, KIT, RET, TIE2, FLT3, VEGF receptor, PDGFR, fibroblast growth factor receptor, EPH receptor kinases	• ≥ 2L in patients for whom no other TKI therapy is indicated • ≥ 2L in patients with T315I mutation [18]	• Incidence ≥ 20% (non-hematologic): abdominal pain, rash, constipation, headache, dry skin, arterial occlusion, fatigue, hypertension, pyrexia, arthralgia, nausea, diarrhea, lipase increased, vomiting, myalgia, and pain in extremity • Most common hematologic: thrombocytopenia, anemia, neutropenia, lymphopenia, leukopenia	• Black-box warning for arterial occlusion, venous thromboembolism, heart failure, and hepatotoxicity (including liver failure and death) • Fetal harm • Hypertension • Pancreatitis • Neuropathy (peripheral and cranial) • Hemorrhage (cerebral and GI) • Ocular toxicity • Fluid retention: peripheral edema, pleural effusion, pericardial effusion, and peripheral swelling • Cardiac arrhythmias • Myelosuppression: thrombocytopenia, neutropenia, anemia • Tumor lysis syndrome • Reversible posterior leukoencephalopathy syndrome • Compromised wound healing and GI perforation

*Based on the current USA labels, for consistency as an overview

Abbreviations: *1L* first-line, *2L* second-line, *AE* adverse event, *ALT* alanine aminotransferase, *AST* aspartate aminotransferase, *CP* chronic-phase, *CML* chronic myeloid leukemia; *CYP* cytochrome P450, *EPH* ephrin receptor, *GI* gastrointestinal, *LV* left ventricular, *MI* myocardial infarction, *PDGFR* platelet-derived growth factor receptor, *TKI* tyrosine kinase inhibitor, *ULN* upper limit of normal, *VEGF* vascular endothelial growth factor

When selecting the most appropriate agent for treatment of CML, the patient’s comorbidities should be considered [12, 19, 20]. Interactions with other therapies would also need to be taken into account, although this consideration alone rarely drives therapy selection. Differences among the TKIs in regard to their inhibitory activity against kinases other than BCR-ABL1 may contribute to their distinct safety profiles [12, 21, 22]. Toxicities frequently observed with TKIs include diarrhea, nausea, vomiting, liver toxicity, rash, musculoskeletal pain, lipase elevation/pancreatitis, myelosuppression, heart failure, QT prolongation, hypertension, thrombosis, peripheral arterial occlusive disease, hyperlipidemia, hyperglycemia, pulmonary hypertension, pneumonitis, and pleural effusion (Table 1) [12].

## Dosing strategies for bosutinib in CP-CML

Both in the USA and the EU, the approved bosutinib starting dose is 400 mg QD in 1L treatment and 500 mg QD in ≥ 2L treatment [1, 2]. Regardless of the dose, bosutinib should be taken with food [1, 2]. Bosutinib is primarily metabolized by cytochrome P-450 (CYP) 3A4, and concomitant use increases bosutinib plasma concentration. Potentially, this could increase the risk of AEs but, as yet, there are no specific studies of AEs directly resulting from drug interactions. Therefore, it is currently recommended that use of bosutinib concomitantly with a strong or moderate CYP3A inhibitor should be avoided [1, 2]. The expert panel considered that when avoidance is not possible or desirable, a careful evaluation of the risk-benefit potential of the concomitant use of such combination should be undertaken, and dose adjustments may be considered.

Upon start of therapy, some patients may experience AEs that may prompt treatment interruptions, dose adjustments, and even early discontinuation of treatment or non-adherence to medication. Some physicians will tailor the starting dose, usually guided by anticipated AEs (in 1L treatment) or AEs on prior TKI therapy (in ≥ 2L treatment), and the panel’s recommendations are discussed below. This is expected to help improve adherence, and it is possible to maintain efficacy at lower doses, as demonstrated by pharmacokinetic studies [1]. Furthermore, the 500 mg dose was approved in ≥ 2L treatment on the basis that this was the dose used in pivotal studies [1] and secondary analyses of both the ≥ 2L phase 1/2 study and 1L treatment in BFORE suggest maintained efficacy even with bosutinib 200 mg QD [23, 24]. At present, no formal studies have been conducted to confirm the minimum effective dose in the 1L or later settings, although an additional study to confirm efficacy is balanced with AE profiles at lower doses is underway in patients receiving ≥ 2L treatment (NCT02906696). However, when a lower starting dose is selected, the aim is generally to bring each patient to the standard dose for their indication [25].

The recommendations for dosing strategies discussed below are based on management of AEs, but it is important to consider the broader clinical setting; in particular, the ability to monitor and manage a patient closely following initiation of treatment is an important consideration regarding starting at the approved dose versus a lower dose [25].

### Recommendations

**Fig. 1:**
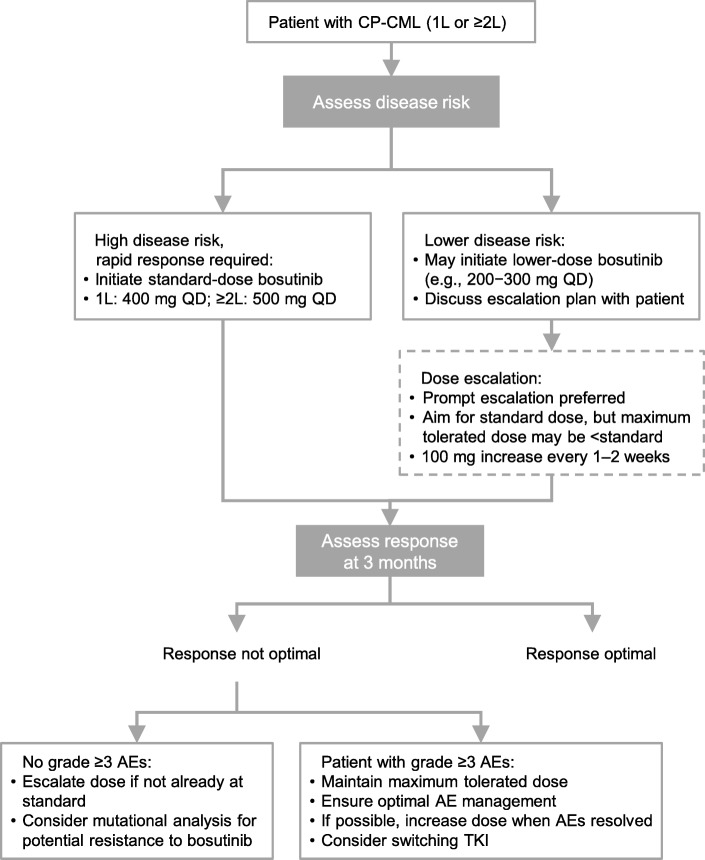
Dosing strategies for bosutinib therapy in CP-CML Abbreviations: AEs, adverse events; CP chronic-phase; CML chronic myeloid leukemia; TKI, tyrosine kinase inhibitor

Figure 1 shows an overview of the panel’s overall recommendations for dosing to manage AEs. In patients previously treated with another TKI inhibitor, the reason for ≥ 2L treatment will frequently affect the selected starting dose; in the case of resistance to a prior TKI inhibitor, a lower dose might be used initially but aiming for rapid escalation to achieve a response. However, in the case of intolerance, a lower dose will typically be used, often without rapid escalation, even though poor tolerability of previous lines does not necessarily predict poor tolerability to bosutinib [7, 8, 26]. Even in instances of intolerance, the status of the disease and the need to maintain efficacy need to be weighted so as not to compromise the probability of an adequate response.

In 1L treatment, the full dose should be initiated in patients with high-risk disease (e.g., high-risk Sokal score) and for whom a rapid response is desired or indicated. Otherwise, a lower dosing strategy with prompt dose escalation is frequently considered. The consensus of the expert panel was that initiating treatment at a lower dose and gradually increasing may be indicated in patients with pre-existing relevant comorbidities or intolerance to previous TKIs and in frail patients, with the aim of reducing the occurrence of AEs, especially during the initial 1–3 months when a lower dose could enable improved compliance and decrease early discontinuation.

In both 1L and ≥ 2L treatment, where tolerability concerns exist, bosutinib may be initiated at 200–300 mg QD. This lower than recommended starting dose should typically be used for no more than 2 to 4 weeks and subsequently increased by 100 mg QD every 1–2 weeks, until the patient reaches either the standard 400-mg QD dose (for 1L treatment) or their maximum tolerated dose, with optimal supportive care. The aim should be to escalate towards the standard dose as soon as possible to optimize efficacy. This dosing strategy needs to be discussed with the patient prior to initiation of treatment, to help ensure adherence to the escalation plan.

If patients do not have an optimal response at 3 months, the probability of a favorable long-term outcome is compromised [27]. Recommendations would depend on specific circumstances but, in general, for a patient receiving 1L treatment, and who does not have grade ≥ 3 AEs, the dose should be escalated. If the dose has already been escalated—typically the case if bosutinib is given as 2L or subsequent line therapy—*BCR-ABL1* mutation analysis is indicated, as recommended by National Comprehensive Cancer Network guidelines [27]. For patients who are on a lower than recommended dose and not having an optimal response at 3 months but who are experiencing grade ≥ 3 AEs, the maximum tolerated dose should be maintained while ensuring opportune and optimal management of AEs, including use of concomitant medications and other supportive care measures to manage toxicity; when the AEs are resolved, the aim should be to increase the dose, if possible. In such cases, the nature of the AEs experienced influences the treatment decisions. For example, many gastrointestinal AEs can be managed with appropriate supportive medications but persistent pleural effusions are difficult to tolerate. Finally, switching to an alternative TKI should also be considered.

Depending on laboratory data, patient history, and the type and severity of the AE, it may be necessary to stop treatment for a short period to allow for resolution of the event, confirm the relationship with the drug, and then reintroduce the TKI. Generally, for events of grade ≥ 2, TKI treatment would be stopped and the patient monitored closely; once the AE resolves, TKI treatment can be re-started. If resolution occurs promptly (e.g., within 2 weeks) and/or spontaneously with no sequelae, treatment may be resumed at the original dose. In many instances, as with most AEs and seen with all TKIs, AEs that occur initially decrease in frequency or intensity or completely resolve over time despite continued therapy. If the AE has not resolved, or if toxicity recurs, the TKI should be stopped until the AE improves (grade < 2), then reintroduced at a lower dose (except in some instances of hematological toxicity). Switching to a different TKI should be considered if the AE is recurrent despite appropriate dose reduction and optimal medical management.

## Recommendations for managing AEs following treatment with bosutinib in patients with CP-CML

Management strategies for the AEs that occur during ≥ 2L bosutinib treatment in patients with CML were discussed in previous publications [12, 28, 29]. Commonly reported AEs that were discussed by the expert group are shown in Table 2, for the 1-year follow-up of the 1L BFORE study and the 4-year follow-up of the ≥ 2L phase 1/2 study.

**Table 2:** Rates of selected AEs in key trials of bosutinib treatment of CP-CML

Patients, safety set	First-line study [6] Phase 3 BFORE trial (NCT02130557)		Subsequent-line study [1] Phase 1/2 study (NCT00261846)	
	CP-CML, newly diagnosed		CP-CML, resistant/intolerant to prior therapy (combined imatinib-only and imatinib plus ≥ 1 additional TKI cohorts)*	
	*n* = 268		*n* = 403	
Follow-up	Minimum 12 months		Minimum 48 months	
Adverse events, % of patients	All grades	Grade ≥ 3	All grades	Grade 3/4
Diarrhea	70	8	85	9
Nausea	35	0	47	1
Vomiting	18	1	37	3
Liver enzyme abnormalities				
ALT increased	31	19	20	8
AST increased	23	10	16	3
Myelosuppression				
Thrombocytopenia	35	14	40	26
Anemia	19	3	27	11
Neutropenia	11	7	18	12
Leukopenia	6	1	10	4

Abbreviations: *ALT* alanine aminotransferase, *AST* aspartate aminotransferase, *CP* chronic-phase, *CML* chronic myeloid leukemia, *TKI* tyrosine kinase inhibitor

*Two hundred eighty-four previously treated with imatinib only and 119 treated with both imatinib and ≥ 1 additional tyrosine kinase inhibitor

### Diarrhea

In both 1L and ≥ 2L treatment, diarrhea is a common AE that occurs soon after initiation of bosutinib [28]. In BFORE, diarrhea of any grade occurred in 70.1% of patients during the first 12 months of the study; however, only a small proportion of patients had grade ≥ 3 events (7.8%) and few patients discontinued treatment due to diarrhea (*n* = 2, 0.7%) [6]. Similarly, in the 2L cohort, 85.6% had diarrhea (9.5% were grade 3/4), with the vast majority (84%) experiencing the first instance of diarrhea during the first year of treatment [7]. Among the subgroup who were previously intolerant to imatinib, only 1% discontinued due to diarrhea, as did 2% of patients previously resistant to imatinib [7]. Overall rates of diarrhea were similar in the ≥ 3L cohort (83.2% overall; 9.2% grade 3/4) [8].

It is important to consider other factors that may contribute to the occurrence of diarrhea. Diarrhea occurrence may correlate with diet [5, 6, 28, 29] and, because the majority of patients treated with bosutinib experience low-grade diarrhea during treatment, dietary advice, and education prior to and during treatment is important (Table 3). Some patients may become partially lactose-intolerant when they have diarrhea; therefore, dairy products should be avoided during an episode of diarrhea. Other food items that may contribute to the occurrence of diarrhea include foods with high fat content and highly condimented or spicy food. Some patients may prefer to take bosutinib at a time of the day when they are at home and have easy access to facilities. Members of the expert panel noted that use of prophylactic anti-diarrheal medication is not usually indicated because it is often not required, particularly when starting at a lower dose, and has potential side effects, including constipation. Anti-diarrheal medication can be prescribed, but patients should be advised to use it only if necessary and to wait until after the second loose bowel movement before considering their use. The consensus of the expert panel was that the decision to provide supportive medication or when to interrupt bosutinib treatment is patient-dependent and should be considered on an individual basis since the effect of any AE on quality of life is subjective. Therefore, it is important to have close communication with the patient during the first few weeks of therapy to help them manage this AE and assist with advice and answering any questions the patient may have.

**Table 3:** Recommendations for the management of AEs before and during bosutinib treatment of CP-CML

AE	Before treatment	During treatment
Diarrhea	• Ensure patients are aware that diarrhea is common, especially during the first few days or weeks of treatment • Patients should be given dietary guidance, as follows: ◦ Avoid spicy or fatty food, caffeine, alcohol, dairy products, and raw fruit and vegetables (except for banana and apple) ◦ Eat low-fiber starchy food and food that is high in sodium and potassium ◦ Eat small meals, snack frequently, avoid very hot or cold food and drink ◦ Re-introduce a balanced diet (high-fiber food, fruit, and vegetables) once diarrhea has resolved • Advise that diarrhea events are self-limiting and typically decrease over time. Patients should not stop taking bosutinib unless discussed with their doctor • Discuss discontinuation of medications that may exacerbate diarrhea (e.g., bulk laxatives, stool softeners, motility-promoting agents) • Advise patients to rehydrate orally (8–12 large glasses of water per day) and avoid supplements that increase gastrointestinal irritation during episodes of diarrhea	• Investigate non-bosutinib-related causes of diarrhea • Initial management strategies should include oral hydration (8–12 large glasses of clear liquid per day containing water, salt, and sugar), and dietary modification • Ensure elderly patients experiencing diarrhea are monitored carefully to ensure adequate hydration and electrolyte balance • Encourage patients to report abdominal pain and consider its underlying cause • For grade ≥ 3 diarrhea, fluid replacement therapy should continue and treatment should be interrupted • Appropriate anti-diarrheal medication should be administered for grade ≥ 3 diarrhea and may also be considered in some patients with lower-grade diarrhea • After grade ≥ 3 diarrhea, treatment may be resumed upon recovery to grade ≤ 1; if clinically appropriate, re-escalation of bosutinib should be considered when the diarrhea is resolved/improved
Nausea and vomiting	• Patients should be advised that nausea and vomiting may occur during treatment and should be reported to physician • Remind patients that bosutinib should be taken with food	• Patients experiencing nausea or vomiting should be advised to try taking bosutinib at a different time of day (although still at a regular time each day, but a patient currently taking bosutinib in the morning may find afternoon or evening better tolerated) • Encourage patients to eat small meals and snack frequently, avoid mixing very hot or very cold food and drink together, and try to eat foods that are gentle on the stomach, possibly giving examples such as bananas, rice, applesauce, and toast • Patients should be encouraged to eat what appeals to them. They should not miss snacks or meals as nausea can worsen with an empty stomach • If nausea and vomiting cannot be managed conservatively, appropriate anti-emetic therapy should be prescribed and used per specific guidance
Liver enzyme abnormalities	• Measure liver function before initiation of bosutinib • Advise patients that other hepatotoxic drugs should be avoided • Reducing alcohol intake may be recommended if a patient is drinking to excess	• Measure liver function every 2–4 weeks for the first 2–3 months after starting bosutinib, and weekly if practical during the first month as this is the period of greatest risk (in 1L treatment, the median time to onset of increased ALT was 32 days and AST was 43 days; in ≥ 2L, median time to onset of increased ALT was 35 days and AST was 33 days [1]). Subsequently, in patients without evidence of hepatotoxicity, measure liver function every 3 months for the first 2 years; in patients with evidence of hepatotoxicity, more frequent monitoring should be employed • Exclude non-bosutinib-related causes of liver function test elevation • Advise patients with liver transaminase elevations ≥ 2.5 × ULN to avoid hepatotoxic substances and alcohol • Interrupt treatment for liver transaminase levels > 5 × ULN. At recovery to ≤ 2.5 × ULN, treatment may be resumed at a lower dose and re-escalation considered if clinically appropriate. If re-escalation occurs, then frequent monitoring should be employed • When recovery takes > 4 weeks and liver function tests do not appear to be improving satisfactorily, discontinuation should be considered • Treatment should be discontinued for liver transaminase levels ≥ 3 × ULN concurrently with bilirubin elevations > 2 × ULN and alkaline phosphatase levels < 2 × ULN
Myelosuppression	• Obtain complete blood counts prior to initiating treatment	• Complete blood counts should be performed weekly for the first month and then once per month thereafter, or as clinically indicated (for example, if patients are established on bosutinib and not returning regularly to the clinic, counts may be less frequent) • In patients with persisting cytopenia, consider: ◦ Modifying starting dose ◦ Using concomitant supportive care to enable continuation of treatment • In patients with advanced disease, treatment interruptions should be minimized and supportive care provided
Skin disorders		• Assess possible causes of rash, e.g., contact with inflammatory substances, allergies, side effect of drugs other than bosutinib • Inform patients that adequate hydration (daily fluid intake of ≥ 2–3 l) will assist in the maintenance of healthy skin • Promote basic skin care, encourage patients to avoid factors that cause skin irritation, use pH-neutral soaps, and wear loose-fitting, lightweight cotton clothes • Manage BCR-ABL1 TKI-induced rashes with antihistamines and topical treatments • If a clinically significant moderate or severe rash develops, consider consultation with a dermatologist for the use of topical or systemic medical treatments
Renal dysfunction	• Renal function status should be measured before initiation of treatment, particularly in patients with pre-existing renal impairment or risk factors for renal dysfunction	• Renal function status should continue to be measured • Patients with risk factors for grade ≥ 3b estimated glomerular filtration rate should be monitored closely • Adjust dose in patients with moderate to severe renal impairment, accompanied by close response monitoring at the reduced dose • Patients should be made aware of the possibility of developing renal problems and advised to immediately report changes in urinary frequency, polyuria, or oliguria
Cardiac events	• Although cardiac events are not common, a patient starting any TKI should have their risk of cardiovascular events assessed • Assess risk factors for arterio-occlusive disease, including hypertension, hyperlipidemia, tobacco use, unhealthy diet, lack of physical activity, etc. and optimize management • Prior to initiating therapy, assessment for risk of QTc prolongation (medical history and use of concomitant medications) and a baseline electrocardiogram are recommended • Hypokalemia or hypomagnesemia must be corrected prior to treatment	• Potassium and magnesium levels should be monitored periodically during therapy • Heart failure was not commonly reported in bosutinib trials, and routine cardiac assessments are not required in all patients. Instead, patients with risk factors should be clinically assessed and additional tests (such as echocardiograms, electrocardiograms) performed as clinically indicated. In patients who develop heart failure, the cancer status should not affect how it is managed: current guidelines for management of heart failure should be followed

Abbreviations: *AE* adverse event, *CP* chronic-phase, *CML* chronic myeloid leukemia, *TKI* tyrosine kinase inhibitor, *ULN* upper limit of normal

Blood in the stool or clinical signs of dehydration would be clear indicators to stop bosutinib in any patient. Members of the expert panel recommended clinicians initiate bosutinib at a lower than recommended dose if there are concerns about gastrointestinal AEs, be proactive in terms of prescribing anti-diarrheal medication, be aware of when drug interruption is recommended, and adapt to the needs of the patient (Table 3). Patients should also be encouraged to report abdominal pain and to keep well hydrated. Importantly, diarrhea typically occurs during the first few weeks of therapy and most commonly improves spontaneously over time despite continued therapy. Therefore, support and education of the patient is important in these early stages to ensure the patient can stay on therapy and have the opportunity to benefit from it.

### Nausea and vomiting

In 1L and 2L bosutinib treatment, nausea and vomiting of any grade were commonly reported, but grade ≥ 3 events were rare (Table 2). In the BFORE trial, nausea and vomiting (all grades) occurred in 35.1% and 17.9% of patients, respectively (similar to proportions observed with imatinib, 38.5% and 16.2%, respectively) [6], whereas in the phase 1/2 study, they occurred in 46.1% and 37.3% of the 2L cohort [7] and in 47.9% and 37.8% of the ≥ 3 L cohort [8].

Patients should be made aware that these AEs are common and that they should report them. Guidance should be given on how to manage food intake to minimize these events, including simple advice such as taking bosutinib with meals, eating small meals and snacking frequently, and patients may also be advised to change the time of day they take bosutinib (Table 3**)**. Anti-emetics may be prescribed if the nausea and vomiting are troublesome, although this is only infrequently needed.

### Liver enzyme abnormalities

Increased ALT and AST were common in the BFORE trial, with increased ALT occurring in a greater proportion of bosutinib-treated patients than increased AST (all grades: 30.6% vs 22.8%, respectively; grade ≥ 3: 19.0% vs 9.7%, respectively) [6]. In ≥ 2L treatment, in the 2L cohort, increased ALT occurred in 22.2% and increased AST in 19.7% [7], whereas in the ≥ 3L cohort, 16% were reported to have either increased ALT or AST [8].

In all patients treated with bosutinib, liver enzyme levels should be monitored regularly (e.g., every 1–2 weeks) and potential causes of elevations not related to bosutinib treatment should be considered (Table 3). Treatment interruption or discontinuation may be necessary depending on the enzyme levels (Table 3). For patients with liver enzyme abnormalities, consultation with a hepatologist should be considered to assess other causes and whether steroids may be beneficial. Patients should be advised to avoid other hepatotoxic drugs and excess alcohol consumption. Liver enzyme elevations usually occur early during the course of therapy, thus monitoring is recommended more frequently during the first few weeks of therapy. Once the patient is deemed stable, the frequency of liver enzyme tests can be decreased. It is not uncommon for early elevations to improve or even resolve spontaneously over time, making early evaluation and management critical to enable the patient to continue therapy, providing the opportunity to achieve a response.

### Myelosuppression

In the BFORE trial, the proportions of bosutinib-treated patients reporting all-grade thrombocytopenia, anemia, neutropenia, and leukopenia were similar to ≥ 2L treatment (Table 2). Proportions of patients with myelosuppression events were also similar between the two cohorts receiving ≥ 2L treatment (2L cohort: thrombocytopenia, 41.5% overall and 25.4% grade 3 or 4; anemia 29.2% overall and 13.4% grade 3 or 4; neutropenia 16.2% overall and 9.9% grade 3 or 4 [7], ≥ 3L cohort: thrombocytopenia, 38.7% overall and 26.1% grade 3 or 4; neutropenia, 21.0% overall and 16.0% grade 3 or 4; anemia, 20.2% overall and 6.7% grade 3 or 4 [8]).

Complete blood counts should be obtained prior to initiating treatment and should continue to be monitored during treatment (Table 3). Treatment interruptions are only recommended for grade ≥ 3 neutropenia (absolute neutrophil count ≤ 1 × 10^9^/L) or thrombocytopenia (platelets ≤ 50 × 10^9^/L). Peripheral blood counts should be monitored at least once weekly thereafter to monitor recovery. If the counts recover within 2 weeks, treatment can be resumed at the same dose; if it takes longer to recover, dose reductions by 100 mg can be implemented. Some patients may tolerate lower peripheral blood counts without the need of interruption or dose adjustment but this should be evaluated on a case-by-case basis. Dose modification and use of concomitant supportive care may enable continuation of treatment. Blood counts often spontaneously return to normal or near-normal levels after a few weeks or once complete cytogenetic response is achieved. When needed, depending on the response achieved and the goals of therapy for the given patient, the dose could be escalated at that point.

### Skin disorders

In the BFORE trial, rashes occurred in 53 (19.8%) bosutinib-treated patients but were typically mild [6]. In the phase 1/2 study, rashes occurred in 36.3% of the 2L cohort and 27.7% of the ≥ 3L cohort [7, 8].

Causes of rash not related to bosutinib treatment should be considered. A careful review of concomitant medications or other possible causes should be undertaken. The importance of hydration and good skin care as well as the elimination of possible irritants or topical allergens should be emphasized (Table 3). Members of the expert panel agreed that consultation with a dermatologist, and early initiation of steroids (topical or systemic) may be useful in treating skin disorders associated with bosutinib.

### Renal dysfunction

Renal AE data has not yet been reported for the BFORE trial [6]. In an analysis of the phase 1/2 study and the BELA trial, renal AEs were reported in 52/403 patients with CP-CML (13%) receiving ≥ 2L bosutinib, and in 22/248 (9%) receiving 1L bosutinib, over a follow-up of at least 48 months [30]. The most common renal AE in both studies was increased blood creatinine (in 10% of the ≥ 2L patients and 6% of the 1L patients) [30]. There is a modest decrease in creatinine clearance in some patients that most frequently occurs early during the course of therapy and stabilizes or improves over time.

The panel recommended that patients should be made aware of the possibility of renal dysfunction and should be advised to report changes in urinary symptoms to their clinician (Table 3). Review of concomitant medications or other nephrotoxic agents, as well as confirming adequate hydration, should be routine during patient evaluation. Renal function should be measured before and during treatment, and bosutinib doses may need to be adjusted [31].

### Other considerations

Pleural effusions occurred in 1.9% of bosutinib-treated patients in the BFORE trial, 10.6% of the 2L cohort, and 16.8% of the ≥ 3L cohort [6–8]. Pericardial effusions and pulmonary edema were less common, but have been observed with bosutinib treatment [1].

Patients who develop signs and symptoms of pleural/pericardial effusions or pulmonary edema during bosutinib treatment should undergo evaluation. Patients should be advised to call their healthcare team when symptoms occur that may signal these AEs. Treatment may be discontinued until effusions improve and then bosutinib should be restarted at a lower dose (for effusions grade ≤ 2). It is rare that patients require thoracentesis or pericardiocentesis.

Cardiac events (QT prolongation, atrial fibrillation, sinus bradycardia, tachycardia, supraventricular tachycardia, bradycardia, premature ventricular contractions, pericardial effusion, right bundle branch block, sinus tachycardia, or premature atrial contractions) were uncommon in the BFORE trial, with overall occurrence in 5.2% of patients receiving bosutinib, of which 0.7% were grade ≥ 3 and considered by the investigator to be drug-related [6]. In the BELA study, for which longer follow-up is available, the exposure-adjusted rate of cardiovascular events in bosutinib-treated patients was not significantly different from that in imatinib-treated patients [32]. All patients starting any TKI should have their risk of cardiovascular events assessed, and members of the expert panel recommended that patients with risk factors be assessed using echocardiograms and/or electrocardiograms, with ongoing monitoring as necessary. For example, prior to initiating bosutinib therapy, assessment for increased risk of corrected QT (QTc) prolongation (medical history and use of concomitant medications) and a baseline electrocardiogram are recommended. QT prolongation occurred in 1.5% of patients receiving bosutinib in the BFORE trial [6]. Grade ≥ 3 QTc prolongation is uncommon with bosutinib (occurring in 0.4% of patients in the BFORE trial) [6]. Hypokalemia or hypomagnesemia must be corrected prior to treatment and monitored periodically during therapy. Traditional cardiovascular risk factors should be assessed in line with general recommendations [33, 34], and management should be optimized both before and during TKI therapy. Management of heart failure should follow current guidelines, which consider heart failure stage [35, 36].

Additionally, members of the expert panel noted that hepatitis B and C status should be checked prior to initiation of any TKI therapy. Patients with active hepatitis or at risk of reactivation should be referred to a hepatologist for further investigation and the viral load should be followed. Of note, treatment with bosutinib is not contraindicated in these patients.

## Conclusions

The BFORE trial demonstrated the efficacy of bosutinib in patients with newly diagnosed CP-CML, and also reported an AE profile similar to that previously seen in patients receiving bosutinib as second-line or subsequent therapy. Concerns around AEs will inevitably need to be taken into account when considering bosutinib as a 1L option, or when switching from another TKI—whether switching due to intolerance of AEs or due to resistance. A number of strategies may be considered to reduce issues with tolerability, with the ultimate aim of adherence to the maximum tolerated dosing. Patient education of goals and known AEs, and optimum communication with patients throughout the course of therapy may help management of AEs, as well as gain their support for dose escalations, when needed, to optimize the efficacy of therapy.

## Abbreviations

1L: First-line
2L: Second-line
AE: Adverse event
ALT: Alanine aminotransferase
AST: Aspartate aminotransferase
BELA: Bosutinib Efficacy and safety in newly diagnosed chronic myeloid LeukemiA
BFORE: Bosutinib trial in First-line chrOnic myelogenous leukemia tREatment
CML: Chronic myeloid leukemia
CP: Chronic-phase
CYP: Cytochrome P450
GI: Gastrointestinal
LV: Left ventricular
MI: Myocardial infarction
PDGFR: Platelet-derived growth factor receptor
QD: Once daily
QTc: Corrected QT
TKI: Tyrosine kinase inhibitor
ULN: Upper limit of normal
VEGF: Vascular endothelial growth factor
